# MicroRNA-381-3p Functions as a Dual Suppressor of Apoptosis and Necroptosis and Promotes Proliferation of Renal Cancer Cells

**DOI:** 10.3389/fcell.2020.00290

**Published:** 2020-04-28

**Authors:** Cong Zhao, Yifei Zhou, Qiao Ran, Ying Yao, Haoran Zhang, Jie Ju, Tao Yang, Wei Zhang, Xiaoliang Yu, Sudan He

**Affiliations:** ^1^State Key Laboratory of Radiation Medicine and Protection, Cyrus Tang Hematology Center and Collaborative Innovation Center of Hematology, Soochow University, Suzhou, China; ^2^Center of Systems Medicine, Institute of Basic Medical Sciences, Chinese Academy of Medical Sciences and Peking Union Medical College, Beijing, China; ^3^Suzhou Institute of Systems Medicine, Suzhou, China

**Keywords:** renal cell carcinoma, miR-381-3p, apoptosis, necroptosis, microRNA

## Abstract

Renal cell carcinoma (RCC) is the most common type of kidney cancer. It has a poor prognosis, with approximately 20–30% of patients developing recurrent and/or metastatic diseases that is relatively high resistant to conventional therapy. Resisting cell death is a hallmark of cancer cells. Apoptosis is a form of programmed cell death mediated by the activation of caspases. Necroptosis is a form of regulated necrosis that relies on the activation of receptor-interacting protein kinase 1 (RIPK1), RIPK3 and mixed lineage kinase domain-like protein (MLKL), the substrate of RIPK3. Cancer cells often display apoptosis resistance via upregulation of anti-apoptotic genes and defective necroptosis due to the epigenetic silence of *Ripk3*. MicroRNAs (miRNAs) are non-coding small RNAs that are involved in numerous biological processes including cell proliferation, differentiation and death. In this study, we screened a set of ∼120 miRNAs for apoptosis-regulating miRNAs and identified miR-381-3p as a suppressor of TNF-induced apoptosis in various cancer cells. Ectopic expression of miR-381-3p inhibits the activation of caspase-8 and caspase-3. The expression level of miR-381-3p inversely correlates with the sensitivity of cancer cells to TNF-induced apoptosis. Moreover, we found that overexpression of miR-381-3p blocks TNF-induced necroptosis by inhibiting the activation of RIPK3 and MLKL. Of note, Kaplan-Meier Plotter analysis demonstrates that papillary RCC patients with high miR-381-3p expression have a lower overall survival than those with low expression level of miR-381-3p. Importantly, miR-381-3p overexpression promotes colony formation in human renal cancer cells. Thus, miR-381-3p acts as an oncogenic miRNA that counteracts both apoptotic and necroptotic signaling pathways. Our findings highlight miR-381-3p as a biomarker for predicting sensitivity to apoptosis and necroptosis, and as a possible therapeutic target for RCC.

## Introduction

In multicellular organisms, cell death is crucial for the individual development and homeostasis maintenance. Apoptosis is a well-defined form of programmed cell death that is characterized with morphological changes including cell shrinkage, chromatin condensation, DNA fragmentation and the formation of membrane-surrounded apoptotic bodies (Kerr et al., 1972). Apoptosis is executed by a series of cysteine proteases called caspases (Thornberry and Lazebnik, 1998). In mammalian cells, apoptosis can be initiated by the activation of the intrinsic mitochondrial pathway or by the extrinsic death receptor pathway. Activation of mitochondria pathway results in the release of mitochondrial cytochrome c to the cytosol and subsequent assembly of the apoptosome, a protein complex comprised of cytochrome c, procaspase-9 and apoptotic protease activating factor-1 (Apaf-1) (Li et al., 1997). This event leads to the activation of caspase-9. Smac/Diablo acts as a pro-apoptotic protein that is released from mitochondria to the cytosol and interacts with inhibitors of apoptosis proteins (IAPs) to relieve IAPs-mediated inhibition of caspases. The extrinsic pathway is initiated by the binding of death ligands of the TNF receptor superfamily, including TNF-α, FasL, and TRAIL, to their respective death receptors: TNFR, Fas and TRAILR (Ashkenazi and Dixit, 1998; Peter and Krammer, 2003). Ligation of TNF to TNFR1 results in formation of a membrane protein complex (called Complex I) consisting of TNFR1, receptor-interacting protein kinases 1 (RIPK1), cIAP1/2, and TRAF2 (Micheau and Tschopp, 2003). RIPK1 is ubiquitinated in Complex I to mediate NF-κB activation (Ea et al., 2006; Li et al., 2006; Wu et al., 2006). Smac mimetic is a small molecule that can mimic the function of Smac protein (Li et al., 2004). In the presence of Smac mimetic, cIAP1/2 are degraded and this process promotes the deubiquitination of RIPK1 by CYLD (Wright et al., 2007; Hitomi et al., 2008). RIPK1 then forms a cytosolic protein complex (Complex II) with FADD and procaspase-8, leading to the activation of caspase-8 (Micheau and Tschopp, 2003; Wang et al., 2008). Activated caspase-9 or caspase-8 can cleave and activate the executor caspases such as caspase-3 and caspase-7, eventually leading to apoptosis.

Necroptosis is a form of regulated necrosis that displays morphological features including cell swelling and membrane rupture. Necroptosis can be induced by activation of the TNFR family of receptors (TNFR1, Fas, TRAILR) (Laster et al., 1988; Holler et al., 2000), TLR3 and TLR4 (He et al., 2011; Kaiser et al., 2013), interferon receptors (IFNRs) (Robinson et al., 2012) and pathogen infection (Nailwal and Chan, 2019). When capase-8 activity is impaired or inhibited by the caspase inhibitor zVAD, TNF-induced apoptosis switches to TNF-induced necroptosis (Laster et al., 1988; Vercammen et al., 1998). RIPK1 interacts with RIPK3 through their RIP homotypic interaction motif (RHIM) domains, leading to the activation of RIPK3 (Cho et al., 2009; He et al., 2009; Zhang et al., 2009). The activated RIPK3 phosphorylates the substrate protein MLKL (Sun et al., 2012; Zhao et al., 2012), followed by its oligomerization and translocation to the plasma membrane, eventually leading to necroptosis (He and Wang, 2018).

Renal cell carcinoma (RCC) accounts for ∼90% of kidney cancers and is one of the most lethal urological cancer. Around 20–30% patients with localized RCC have a recurrence and/or develop metastatic disease that is relatively high resistant to conventional chemotherapy and radiotherapy (Pantuck et al., 2001). Resisting cell death is considered as a hallmark of cancer cells. Cancer cells often display increased expression of anti-apoptotic genes, including IAPs and anti-apoptotic Bcl-2 family, which is associated with increased resistance of cancer cells to apoptotic stimuli (Pfeffer and Singh, 2018). Many cancer cells exert a defect in necroptosis due to epigenetic silence of *Ripk3* gene. MicroRNAs (miRNAs) are a type of small endogenous single-stranded non-coding RNAs that negatively regulate the expression of target genes by binding to their 3′-UTR region. Increasing evidence suggests that miRNAs are involved in the regulation of various biological processes, including cell proliferation, differentiation, and cell death (Negrini et al., 2009). Studies have shown that some microRNAs are involved in regulating apoptotic pathway in cancer cells (Su et al., 2015; Shirjang et al., 2020). For example, miR-187, miR-181c and miR-34a target TNF-α, leading to suppression of TNF-induced apoptosis (Rossato et al., 2012; Zhang et al., 2012; Guennewig et al., 2014). MiR-708 and miR-22 are downregulated in RCC samples. The overexpression of miR-708 induces apoptosis and suppresses clonogenicity in renal cancer cells (Saini et al., 2011). MiR-22 overexpression increases acetylated p53 and apoptosis by reducing the expression of SIRT1 (Zhang et al., 2016). Additionally, miR-155 inhibits necroptosis in human cardiomyocyte progenitor cells through targeting RIPK1 (Liu et al., 2011). Therefore, identification of miRNAs regulating apoptosis and necroptosis could offer new insights into exploring biomarkers or therapeutic targets for cancer.

In the present study, we discovered miR-381-3p as a dual suppressor of TNF-induced apoptosis and necroptosis in multiple cancer cells. MiR-381-3p interferes with TNF-induced apoptosis by inhibiting the activation of caspase-8 and caspase-3. In addition, miR-381-3p negatively regulates TNF-induced necroptosis through inhibiting the activation of RIPK3 and MLKL. Notably, Kaplan-Meier Plotter analysis has shown that RCC patients with high miR-381-3p expression correlates with a lower overall survival. Remarkably, miR-381-3p overexpression promotes cell proliferation and colony formation of human renal cancer cells.

## Materials and Methods

### Cell Culture

HT-29, OSRC-2, 786, Panc-1, MKN45, and HEK-293T cells were from ATCC. RKO, SW480 and SW620 were kindly provided by Dr. Jianming Li (Soochow University). These cells were cultured in DMEM medium (Invitrogen) supplemented with 10% fetal bovine serum (Invitrogen) and 100 units/mL Penicillin-Streptomycin-Glutamine (Hyclone) in a humidified incubator at 37°C and 5% CO_2_. HT-29 stably expressing Flag-RIPK3 was cultured in complete medium containing 2 μg/ml G418 (Calbiochem) as previously described (He et al., 2009).

### Cell Viability Assay

Cells were seeded in 96-well plates and then treated as indicated. The cell viability was analyzed by using the Cell Titer-Glo Luminescent Cell Viability Assay kit (Promega, United States) according to the manufacturer’s instructions.

### Reagents and Antibodies

TNF-α recombinant protein was generated as previously described (Wang et al., 2008). The Smac mimetic compound was kindly provided by Dr. Xiaodong Wang (National Institute of Biological Sciences, Beijing). z-VAD was bought from Bachem (Babendorf, Switzerland). The following antibodies were used: hRIPK1 (BD Biosciences, 610458), p-hRIPK1 (CST, 65746), p-hRIPK3 (Abcam, 209384), p-hMLKL (Abcam, 187091), caspase-8 (CST, 9746), caspase-3 (CST, 9665), cleaved-caspase-3 (CST, 9664), PARP (CST, 9542), FADD (Abcam, 52935), TNFR1 (CST, 3736), TRADD (CST, 3684), TRAF2 (CST, 4712), p-IκB-α (CST, 9246), CYLD (CST, 4495), β-actin (Sigma, A2066). The antibodies recognizing human RIPK3 and MLKL were generated against full-length human recombination proteins.

### MicroRNA Screening

Around 120 microRNAs were synthesized by GenePharma Co., Ltd. (Shanghai, China). MicroRNAs were diluted in Opti-MEM medium (Invitrogen, United States) and then transferred into 96-well plates. Lipo2000 was diluted in Opti-MEM medium and incubated for 5 min, then were added to those 96-well plates. After incubation for 20 min, Panc-1 cells were added into the plates at density of 3 × 10^3^ cells per well. Forty-eight hours (h) after transfection, cells were treated with PBS or TNF-α/Smac mimetic for 24 h, followed by cell viability analysis. The negative control oligo (miR-NC) and a RIPK1 siRNA oligo were used as negative control and positive control, respectively.

### SiRNA Transfection

The siRNA oligos were transfected into cells using Lipofectamine 2000 (Invitrogen, United States) according to the manufacturer’s instructions. The siRNA oligos were purchased from GenePharma Co., Ltd. (Shanghai, China). The following siRNA oligos were used:

**Table:** 

Oligo	Sequence (5′→3′)
miR-NC	AACGUACGCGGAAUACUUCGA
miR-381-3p	UAUACAAGGGCAAGCUCUCUGU
si-hCYLD	AAGGGTAGAACCTTTGCTAAA
si-hRIPK1	CCACTAGTCTGACGGATAA
si-hRIPK3	GCUACGAUGUGGCGGUCAA
si-hMLKL	CAAACTTCCTGGTAACTCA
	

### Western Blot Analysis

Cell pellet was collected by centrifugation at 13000 × *g* for 1 min and resuspended in lysis buffer [20 mM Tris–HCl, pH 7.4, 150 mM NaCl, 10% glycerol, 1% Triton X-100, 1 mM Na_3_VO_4_, 25 mM β-glycerol phosphate, 0.1 mM PMSF, a complete protease inhibitor set (Roche)]. Cell lysate was incubated on ice for 20 min, and then centrifuged at 13000 × *g* for 20 min at 4°C. The supernatants were collected and subjected to further western blot analysis.

### Real-Time Quantitative PCR Analysis

Total RNA was extracted from cells using Trizol Reagent (Invitrogen, United States) according to the manufacturer’s instructions. RNA was reversely transcribed into cDNA using HiScript II Q RT SuperMix (Vazyme, China). The gene expression was determined by quantitative real time PCR using SYBR Green Master Mix (Biotool, United States) performed in a Roche LightCycler 480 II system. The following primers were used:

**Table:** 

Gene	Sequence (5′→3′)
miR-381-3p	F: AAAGCGAGGTTGCCCTTTGT
	R: TACTCACAGAGAGCTTGCCC
U6	F: CTCGCTTCGGCAGCACA
	R: AACGCTTCACGAATTTGCGT
TNFR1	F: TGCCAGGAGAAACAGAACAC
	R: TCCTCAGTGCCCTTAACATTC
TRADD	F: GCTGTTTGAGTTGCATCCTAGC
	R: CCGCACTTCAGATTTCGCA
TRAF2	F: TCCCTGGAGTTGCTACAGC
	R: AGGCGGAGCACAGGTACTT
RIPK1	F: TGGGCGTCATCATAGAGGAAG
	R: CGCCTTTTCCATGTAAGTAGCA
GAPDH	F: GGAGCGAGATCCCTCCAAAAT
	R: GGCTGTTGTCATACTTCTCATGG
	

mRNA and miRNA expression levels were normalized against GAPDH and endogenous U6 small nuclear RNA (U6 snRNA), respectively.

### Flow Cytometry Analysis

The Annexin V-FITC/PI apoptosis detection kit (BD Biosciences, United States) was used according to the manufacturer’s instructions. Panc-1 cells were transfected with miR-NC, miR-381-3p or a RIPK1 siRNA oligo. After 60 h, cells were treated with TNF-α/Smac mimetic. Twenty-four hours later, cells were harvested, then washed with cold PBS and resuspended in staining buffer containing Annexin V- FITC. Then cells were incubated in the dark at room temperature for 20 min, followed by PI staining. Flow cytometry analysis was acquired on a Gallios Flow Cytometer (Beckman Coulter, United States) and data was then analyzed with software Kaluza Analysis.

### Dual-Luciferase Reporter Assay

3′-UTR of human CYLD (hCYLD) was cloned into the pmir-GLO vector (Promega, United States). The recombinant reporter plasmids were validated by DNA sequencing. CYLD-3′UTR reporter plasmid was co-transfected with miR-NC or miR-381-3p into HEK-293T cells. Then cells were harvested in reporter lysis buffer. The luciferase activity was measured 48 h after transfection by Dual-Luciferase Reporter Assay System (Promega, United States). Luciferase activity was normalized to Renilla luciferase activity.

### Colony Formation Assay

OSRC-2 and Panc-1 cells were transfected with miR-NC, miR-381-3p or siRIPK1 in 12-well plates. Forty-eight hours later, cells (8 × 10^2^/well) were reseeded in 6-well plates, and culture media were replaced every 3 days. After around 7 days, cell colonies reached desirable size. Cells were fixed with 10% formalin and stained with Gimasa [Nanjing Jiancheng Chemical Industrial Co., Ltd. (Nanjing, China)] for counting. The total area of OSRC-2 cell colony formation was calculated using Image J software.

### Overall Survival Analysis

The dataset of a total 290 patients with papillary RCC were obtained from the online database Kaplan-Meier Plotter^1^. The overall survival (OS) was analyzed with Kaplan-Meier Plotter and GraphPad Prism.

### Statistical Analysis

Data of cell survival rate are represented as the mean ± standard deviation of triplicates. Significance was evaluated using *t*-tests of GraphPad Prism software. *P*-values were determined by paired-samples *t*-tests. ^∗^*P* < 0.05, ^∗∗^*P* < 0.01, ^∗∗∗^*P* < 0.001.

## Results

### Identification of MiR-381-3p as a Suppressor of TNF-Induced Apoptosis

**FIGURE 1:**
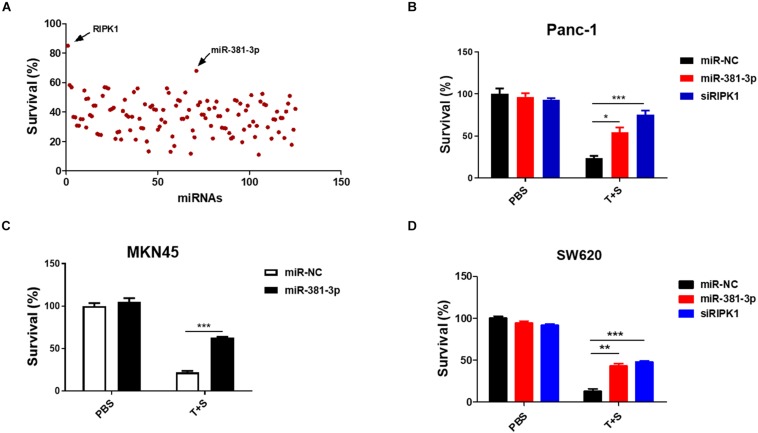
MiR-381-3p inhibited TNF-induced apoptosis in human cancer cells. **(A)** Panc-1 cells cultured in 96-well plates were transfected with miRNAs from a small library of ∼120 miRNAs for 48 h, and subsequently treated with the control PBS or TNF-α (40 ng/ml) plus Smac mimetic (100 nM) (T + S) for 24 h. Cell viability was determined by measuring ATP levels. T: TNF-α; S: Smac mimetic. **(B–D)** Panc-1, MKN45 and SW620 cells were transfected with miR-381-3p for 60 h prior to the treatment with TNF-α plus Smac mimetic for 24 h, and then cell viability was determined. Identical concentrations of TNF-α and Smac mimetic were used in later experiments unless otherwise stated. *P < 0.05, **P < 0.01, ***P < 0.001.

Apoptosis is a highly regulated process of cell death executed by caspases. To identify miRNAs involved in the cellular response to apoptosis, we screened a set of ∼ 120 miRNAs to identify candidate miRNAs regulating TNF-induced apoptosis, which is known to be induced by the treatment of TNF-α plus Smac mimetic (Wang et al., 2008). This screening was carried out in human pancreatic cancer Panc-1 cells, which are known to be sensitive to TNF-α/Smac mimetic. Panc-1 cells were transfected with these miRNAs for 48 h, and then were treated with TNF-a plus Smac mimetic. After 24 h, cell viability was determined by measuring the ATP levels. MiR-381-3p came out as one of the most effective hits that significantly suppressed TNF-induced apoptosis (Figure 1A). A siRNA oligo targeting RIPK1 was used as the positive control (Figure 1A). Further, we confirmed that miR-381-3p efficiently inhibited TNF-induced apoptosis in multiple human cancer cell lines including pancreatic cancer Panc-1 cells, gastric carcinoma MKN45 cells, and colon cancer SW620 cells (Figures 1B–D).

### MiR-381-3p Negatively Regulates Apoptosis by Suppressing Caspase-3 Activation

**FIGURE 2:**
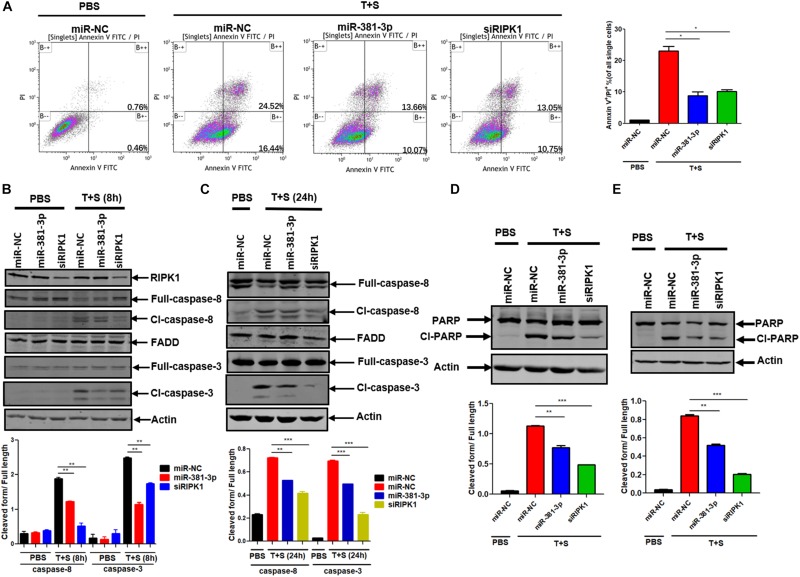
MiR-381-3p negatively regulates apoptosis by suppressing caspase-3 activation. **(A)** Panc-1 cells were transfected with miR-NC, miR-381-3p or siRIPK1 for 60 h, respectively, and then were stained with Annexin V/PI for flow cytometric analysis of apoptosis. **(B,C)** Panc-1 cells were transfected with indicated miRNAs for 60 h and then treated with control PBS or TNF-α plus Smac mimetic for the indicated time. Cell lysates were collected, and aliquots of 40 μg were subjected to western blot analysis of caspase-8, FADD and caspase-3. The ratio of the cleaved form to full-length caspase-8 or caspase-3 was determined by software Image J (lower panel). **(D,E)** Panc-1 and MKN45 cells transfected with miR-381-3p were treated with TNF-α plus Smac mimetic, and analyzed by western blot for PARP cleavage. The ratio of the cleaved form to full-length PARP was also determined by software Image J (lower panel). Cl-caspase-8: cleaved caspase-8. Full caspase-8: full length caspase-8. *P < 0.05, **P < 0.01, ***P < 0.001.

Since miR-381-3p has the ability to inhibit TNF-induced apoptosis, we sought to evaluate the effect of miR-381-3p on the Annexin V-positive cells and caspase activation. Annexin V staining is widely used for the detection of apoptotic cells with the exposure of phosphatidylserine at the outer surface of cell membrane. We transfected miR-381-3p into Panc-1 cells, and treated cells with PBS (as control) or TNF-α/Smac mimetic. The cells were stained with Annexin V and PI, followed by flow cytometry analysis. As shown in Figure 2A, transfection of miR-381-3p reduced the percentage of both Annexin V and PI positive cells in response to the treatment of TNF-α/Smac mimetic. As TNF-induced apoptosis is mediated via the activation of a series of caspases including caspase-8, an initiator caspase, and caspase-3, an executioner caspase, we next examined the effect of miR-381-3p on the activation of caspase-8 and caspase-3 by measuring their proteolytic cleavage. As shown in Figures 2B,C, miR-381-3p transfection reduced the cleavage of caspase-8 and the cleavage of caspase-3. Poly (ADP-ribose) polymerase (PARP) is a well-known substrate that is cleaved by activated caspases-3 during apoptosis. We found that miR-381-3p transfection inhibited the cleavage of PARP in both Panc-1 and MKN45 cells treated with TNF-α/Smac mimetic (Figures 2D,E). MiR-381-3p overexpression did not affect the expression levels of caspase-8, FADD and caspase-3 under normal culture conditions (Figure 2B), suggesting that miR-381-3p negatively regulates caspase-8 and caspase-3 activation, but not their expression levels. Collectively, these results demonstrate that miR-381-3p is capable of inhibiting apoptosis by interfering with caspase-8 and caspase-3 activation.

### High Expression of MiR-381-3p Is Correlated With Resistance of Cancer Cells to Apoptosis and Poor Prognosis of RCC Patients

**FIGURE 3:**
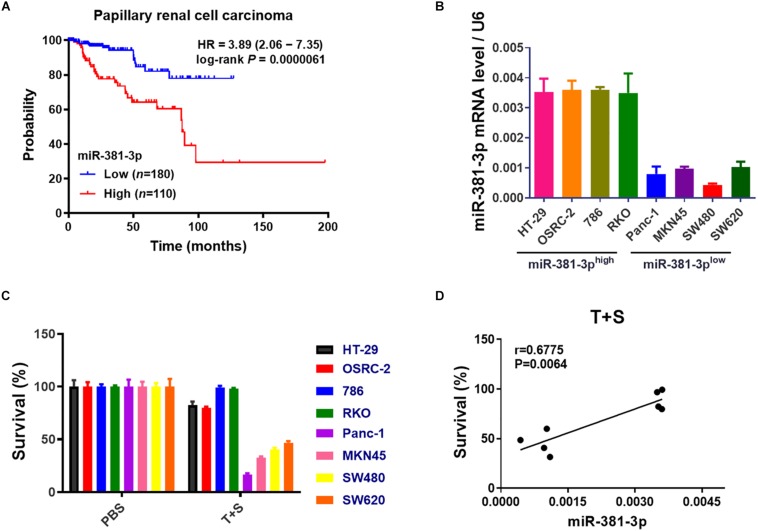
High expression of miR-381-3p is correlated with resistance of cancer cells to apoptosis and poor prognosis of RCC patients. **(A)** The overall survival of papillary RCC patients was compared between individuals with high or low level of miR-381-3p. **(B)** Detection of miR-381-3p mRNA expression level in different human cancer cells by qRT-PCR analysis. U6 was used as an internal control. **(C)** Human cancer cells were treated with the control PBS or TNF-α/Smac mimetic for 24 h. Cell viability was determined by measuring ATP levels. **(D)** Correlation analysis revealed a positive correlation between relative miR-381-3p mRNA expression levels in human cancer cells and survival rate of cells treated with TNF-α/Smac mimetic.

Apoptosis resistance is a hallmark of cancer cells. Having shown that miR-381-3p intervenes with the apoptosis pathway, we sought to examine the prognostic value of miR-381-3p expression in human patients with cancer using the online database Kaplan-Meier Plotter (see text footnote 1). Kaplan–Meier overall survival analysis showed that papillary RCC patients with high miR-381-3p expression level had a significantly shorter overall survival time than those patients with low miR-381-3p expression level (Figure 3A). Considering that miR-381-3p overexpression in both Panc-1 and MKN45 cells enhanced cell resistance to TNF-induced apoptosis, we hypothesized that there is a correlation between the miR-381-3p level and the sensitivity of cancer cells to TNF-induced apoptosis. We measured the expression levels of miR-381-3p mRNA in various human cancer cell lines including human renal cancer cell lines (OSRC-2 and 786), human colon cancer cell lines (HT-29, RKO and SW480 and SW620) in addition to Panc-1 and MKN45. Based on the relative expression level of miR-381-3p, we divided these cell lines into two groups: miR-381-3p high expression cell lines (HT-29, OSRC-2, 786, RKO) and miR-381-3p low expression cell lines (Panc-1, MKN45, SW480, and SW620) (Figure 3B). Notably, the miR-381-3p^*high*^ cells (HT-29, OSRC-2, 786, and RKO), displayed relatively lower sensitivity to TNF-induced apoptosis, compared with the miR-381-3p^*low*^ cells (Panc-1, MKN45, SW480, and SW620) (Figure 3C). Further correlation analysis showed a positive correlation between miR-381-3p expression level and resistance of cells to TNF-induced apoptosis (Figure 3D). Taken together, these results suggest that cellular miR-381-3p expression level in human cancer cell is negatively correlated with cell sensitivity to TNF-induced apoptosis.

### MiR-381-3p Inhibits TNF-Induced Necroptosis

**FIGURE 4:**
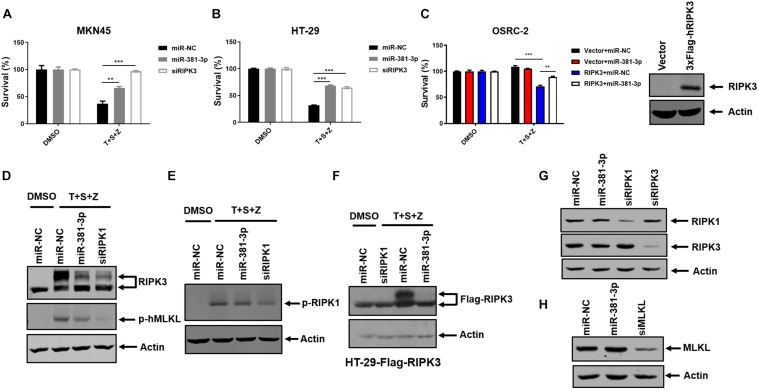
MiR-381-3p inhibits TNF-induced necroptosis. **(A,B)** MKN45 and HT-29 cells were transfected with miR-381-3p for 60 h, and then were treated with the control DMSO or TNFα/Smac mimetic/z-VAD (20 μM) (T + S + Z) for indicated time. Cell viability was determined by measuring ATP levels. Z: z-VAD.**(C)** OSRC-2 cells were transfected with empty vector or human RIPK3 plasmid together with miR-NC or miR-381-3p. After 48 h, cells were treated with TNFα/Smac mimetic/z-VAD for 24 h. Cell viability was determined by measuring ATP levels. OSRC-2 cells were transfected with empty vector or human RIPK3 plasmid for 48 h, then the cell lysates were subjected to western blot analysis of RIPK3. **(D,E)** MiR-NC, miR-381-3p or siRIPK1 was transfected into HT-29 cells for 60 h, then treated with TNFα/Smac mimetic/z-VAD. Cell lysates were collected and subjected to western blot analysis of phos-RIPK3, phos-MLKL and phos-RIPK1. **(F)** HT-29 cells stably expressing Flag-RIPK3 (HT-29-Flag-RIPK3) were transfected with miR-NC, miR-381-3p or siRIPK1 for 60 h, followed by treatment as indicated. Cell lysates were collected and subjected to western blot analysis of Flag-RIPK3 level. **(G,H)** HT-29 cells were transfected with indicated miRNAs for 60 h. The cell lysates were subjected to western blot analysis of RIPK1, RIPK3, and MLKL levels. **P < 0.01, ***P < 0.001.

In addition to apoptosis, TNF is a classical trigger of necroptosis, which is highly regulated by RIPK1, RIPK3, and MLKL. To evaluate the effect of miR-381-3p on TNF-induced necroptosis, both MKN45 and HT-29 cells were transfected with miR-NC or miR-381-3p, followed by treatment with necroptotic stimuli (TNF-α, Smac mimetic and z-VAD), which is known to induce TNF-mediated necroptosis (He et al., 2009). As shown in Figures 4A,B, overexpression of miR-381-3p in MKN45 and HT-29 cells resulted in reduced cell death, suggesting that miR-381-3p suppressed TNF-induced necroptosis. As OSRC-2 cells do not express RIPK3, we further evaluated the impact of miR-381-3p on necroptosis in OSRC-2 cells with ectopic expression of human RIPK3. Ectopic expression of RIPK3 in OSRC-2 cells resulted in death of around 30% cells upon necroptotic stimuli, while this effect was attenuated by miR-381-3p overexpression (Figure 4C). During TNF-induced necroptosis, activation of RIPK1 and RIPK3 leads to the phosphorylation of RIPK1 and RIPK3. The activated RIPK3 phosphorylates the substrate MLKL. We therefore examined the effect of miR-381-3p on the activation of RIPK1, RIPK3, and MLKL. MiR-381-3p transfection decreased the phosphorylation levels of both RIPK3 and MLKL in HT-29 cells treated with TNF-α/Smac mimetic/z-VAD, while overexpression of miR-381-3p had no effect on RIPK1 phosphorylation (Figures 4D,E). We further confirmed the inhibitory effect of miR-381-3p overexpression on RIPK3 phosphorylation in HT-29 cells which stably expressed RIPK3 with a 3X FLAG tag at the N-terminus (Figure 4F). These results demonstrate that miR-381-3p negatively regulates activation of RIPK3 and MLKL acting downstream of RIPK1 activation. To clarify whether miR-381-3p directly targets these key molecules in TNF-induced necroptosis, we evaluated the effect of miR-381-3p on the protein levels of RIPK1, RIPK3, and MLKL. It came out that the expression levels of these three proteins were not affected by miR-381-3p overexpression (Figures 4G,H), suggesting that miR-381-3p does not downregulate the levels of RIPK1, RIPK3, and MLKL. Taken together, miR-381-3p exerts an inhibitory effect on RIPK3 activation and necroptosis.

### MiR-381-3p Does Not Directly Downregulate the Known Common Molecules Involved in TNF-Induced NF-κB Signaling and Cell Death

**FIGURE 5:**
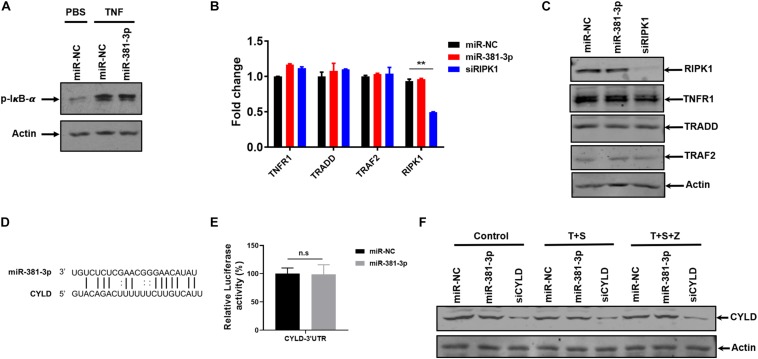
MiR-381-3p does not directly downregulate the known common molecules involved in TNF-induced NF-κB signaling and cell death. **(A)** HT-29 cells were transfected with miR-NC or miR-381-3p for 60 h and then treated with the control PBS or TNF-α for 10 min, phos-IκB-α protein level was detected by western blot. **(B)** HT-29 cells were transfected with miR-NC or miR-381-3p for 60 h, and mRNA levels of *Tnfr1*, *Tradd*, *Traf2*, and *Ripk1* were determined via qRT-PCR. **(C)** HT-29 cells were transfected with miR-NC or miR-381-3p for 60 h. Cell lysates were collected and subjected to western blot analysis of TNFR1, TRADD, TRAF2, and RIPK1. **(D)** MiR-381-3p and its putative binding sequence in the 3′-UTR of CYLD. **(E)** MiR-NC or miR-381-3p was co-transfected with pmir-GlO-CYLD-3′UTR into HEK-293T cells, and luciferase activity was measured. **(F)** HT-29 cells were transfected with miR-381-3p for 60 h, and then were treated with the control PBS, TNF-α/Smac mimetic or TNFα/Smac mimetic/z-VAD for 6 h. Cell lysates were collected and subjected to western blot analysis of CYLD protein level. **P < 0.01.

TNF is a pleiotropic cytokine that can activate NF-κB activation in addition to apoptosis and necroptosis. To assess the effect of miR-381-3p on TNF-induced NF-κB activation, we examined the effect of miR-381-3p on the level of IκB-α phosphorylation, which is a key step in NF-κB activation. As shown in Figure 5A, overexpression of miR-381-3p had no effect on the IκB-α phosphorylation, suggesting that miR-381-3p did not influence IκB-α phosphorylation in response to TNF-α receptor activation. Moreover, miR-381-3p exerted no impacts on the mRNA expression levels of molecules involved in the TNFR complex including TNFR1, TRADD, TRAF2, and RIPK1 (Figure 5B). Given that miRNAs may regulate gene expression through suppressing mRNA translation, we further examined whether the protein levels of these genes in the TNFR complex were affected by miR-381-3p overexpression. The result showed that miR-381-3p had no effect on the protein levels of TNFR1, TRADD, TRAF2, and RIPK1 (Figure 5C). To further investigate possible target of miR-381-3p in the regulation of apoptosis and necroptosis, we searched the miRNA database (microRNA), and found a putative miR-381-3p-binding site located within the 3′-UTR of CYLD (Cylindromatosis) (Figure 5D). CYLD is a de-ubiquitin enzyme which removes ubiquitin chains on RIPK1 to facilitate both TNF-induced apoptosis and necroptosis (Wright et al., 2007). To evaluate whether CYLD is a direct target of miR-381-3p, we generated luciferase report gene of 3′-UTR of CYLD containing the predicted miR-381-3p-binding site. We found that transfection of miR-381-3p did not affect the luciferase signal of 3′-UTR of CYLD (Figure 5E). Moreover, overexpression of miR-381-3p had no obvious impact on CYLD protein level when cells were either cultured under normal condition or treated with apoptotic or necroptotic stimuli (Figure 5F). Collectively, these results suggest that miR-381-3p does not directly downregulate the known common molecules involved in TNF-induced NF-κB signaling, apoptosis and necroptosis.

### MiR-381-3p Promotes Human Renal Cancer Cell Growth and Colony Formation

**FIGURE 6:**
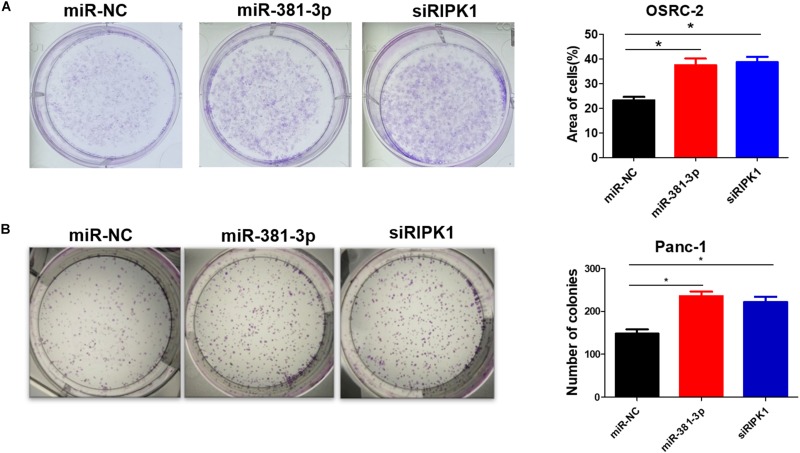
MiR-381-3p promotes human renal cancer cell growth and colony formation. **(A)** Human renal cancer OSRC-2 cells were transfected with miR-NC, miR-381-3p or RIPK1 siRNA oligo, followed by colony formation assay. **(B)** Panc-1 cells were transfected with miR-NC, miR-381-3p or RIPK1 siRNA oligo and then colony formation assay was performed. *P < 0.05.

High expression of miR-381-3p not only enhanced resistance of renal cancer cells to apoptosis and necroptosis (Figures 1, 4), but also was highly correlated with poor prognosis of RCC patients (Figure 3A). Therefore, we speculated that miR-381-3p might contribute to cell proliferation and clonogenicity in renal cancer cells. We further explore the effect of miR-381-3p on colony formation of human renal carcinoma cells. As shown in Figure 6A, miR-381-3p overexpression significantly increased OSRC-2 cell colony formation. Consistently, miR-381-3p overexpression promoted the colony formation in Panc-1 cells (Figure 6B). These results suggest that miR-381-3p promotes survival and proliferation of cancer cells including renal cancer cells.

## Discussion

Cancer cells escape from immunosurveillance by developing strategies to avoid apoptotic and necrotic cell death. The ability of cancer cells to evade cell death usually limits the efficacy of anticancer therapies. Emerging evidence has suggested that miRNAs play important roles as oncogenes or tumor suppressors in various cancers including RCC. In this study, we identify miR-381-3p as a dual suppressor of apoptosis and necroptosis with an oncogenic role in renal cell cancer.

MiR-381-3p has been demonstrated as either oncogenic or tumor suppressive miRNA in various tumor types depending on the target mRNAs. For instance, miR-381-3p overexpression promotes cancer cell proliferation in glioblastoma cells and osteosarcoma cells by targeting the brain relative specific expression gene LRRC4 (Tang et al., 2011; Li et al., 2016). On the contrary, miR-381-3p functions as a tumor suppressor gene in rectal cancer and prostate cancer through suppression of UBE2C (Zhang et al., 2018; Hu et al., 2019). Through a cell-based screening for miRNAs regulating apoptosis, we found that miR-381-3p negatively regulates TNF-induced apoptosis in multiple human cancer cell lines. MiR-381-3p overexpression inhibits the activation of caspase-8 and caspase-3 (Figures 2B,C). In addition, miR-381-3p blocks TNF-induced necroptosis through suppressing activation of RIPK3 and MLKL, whereas it has no impact on RIPK1 phosphorylation (Figures 4C–E). Taken together, our study reveals that miR-381-3p plays a crucial role in counteracting both apoptosis and necroptosis in cancer cells.

Considering that miR-381-3p is a dual suppressor of apoptosis and necroptosis, we speculated that it may target common molecules involved in apoptotic and necroptotic cell death. In fact, miR-381-3p overexpression does not affect the expression levels of RIPK1 and CYLD (Figures 4F, 5E). Of note, miR-381-3p specifically inhibits TNF-induced apoptosis and necroptosis, while it has no effect on TNF-induced NF-κB activation (Figure 5A), suggesting that molecules involved in the TNFR complex are not the functional targets of miR-381-3p. Consistently, miR-381-3p does not downregulate the mRNA and protein levels of molecules in the TNFR complex including TNFR1, TRADD, TRAF2, and RIPK1 (Figures 5B,C). Future studies will be required to explore the direct targets of miR-381-3p in regulating TNF-induced apoptosis and necroptosis.

Our study demonstrates that miR-381-3p expression varies among different types of human cancer cell lines, which negatively correlates with tumor cell sensitivity to TNF-induced apoptosis (Figures 3B–D). MiR-381-3p expression is relatively high in human renal carcinoma cells. Remarkably, papillary RCC patients with high expression level of miR-381-3p is associated with a poor prognosis (Figure 3A). Indeed, miR-381-3p overexpression promotes clonogenicity of human renal cancer cells (Figure 6A). Thus, miR-381-3p could be a biomarker for predicting sensitivity to apoptosis and necroptosis, and may be a potential target for renal cancer therapy.

## Data Availability Statement

All datasets generated for this study are included in the article/supplementary material.

## Author Contributions

SH and XY designed this study and revised the manuscript. CZ and YZ performed the molecular biology studies and colony formation, analyzed the data, and drafted the manuscript. QR, YY, HZ, and JJ performed cell culture and qRT-PCR. TY and WZ performed flow cytometry analysis and plasmid construction.

## Conflict of Interest

The authors declare that the research was conducted in the absence of any commercial or financial relationships that could be construed as a potential conflict of interest.

## Footnotes

1 http://kmplot.com/analysis/
